# Effects of Heshouwuyin on gene expression of the insulin/IGF signalling pathway in rat testis and spermatogenic cells

**DOI:** 10.1080/13880209.2020.1839511

**Published:** 2020-12-02

**Authors:** Hongjie Wang, Boying Shan, Yulei Duan, Juan Zhu, Liping Jiang, Yang Liu, Yan Zhang, Feng Qi, Siyun Niu

**Affiliations:** a School of Medicine, Hebei University, Baoding, China; b The Department of Internal Medicine, Baoding No.1 Hospital, Baoding, China

**Keywords:** Male reproduction, senescence, INSR, IRS1, IRS2, IGF1, IGFBP3

## Abstract

**Context:**

The Chinese herbal formula Heshouwu decoction (Heshouwuyin) has protective effects on testicular function in aging male rats, but the mechanism is unknown.

**Objective:**

This study investigated whether Heshouwuyin affects the testicular function of aging rats by regulating the insulin/IGF signalling pathway.

**Materials and methods:**

Sixteen-month-old male Wistar rats in the Heshouwuyin group and the natural-aging group were orally administered Heshouwuyin granules (0.056 g/kg) or equivalent normal saline for 60 d. The testicular tissue of 12-month-old male Wistar rats was removed as a young control group (*n* = 10). The testicular tissue and spermatogenic cells were studied.

**Results:**

The immunofluorescence results revealed that the insulin receptor (INSR)- (0.056 ± 0.00548), insulin receptor substrate 1(IRS1)- (0.251 ± 0.031), IRS2 (0.230 ± 0.019)- and insulin-like growth factor 1 (IGF1)-positive cell rate (0.33 ± 0.04) in the aging group was higher than that in the young control group (0.116 ± 0.011, 0.401 ± 0.0256, 0.427 ± 0.031, 0.56 ± 0.031; *p* < 0.01), and the IGF-binding protein 3 (IGFBP3)-positive cell rate (0.42 ± 0.024) was lower than that (0.06 ± 0.027) in the young group (*p* < 0.01). The intervention of Heshouwuyin reversed the above phenomena. The qPCR and immunoblot results were consistent with those of the immunofluorescence. The same results were obtained in spermatogenic cells.

**Conclusions:**

Our research shows that Heshouwuyin can regulate the insulin/IGF signalling pathway to improve testicular function, and provides an experimental basis for further clinical use.

## Introduction

Aging is accompanied by the occurrence of various aging-related conditions, and the organism directly or indirectly regulates aging by altering the levels of sex hormones (Decaroli and Rochira 2017). The testes are important organs that secrete male sex hormones such as testosterone. After the age of 60, the testicles degenerate, and the secretion of sex hormones is reduced. These changes result in decreased fertility and related diseases such as diabetes, hypertension, osteoporosis, and senile dementia (Salminen et al. 2015; Cawthon et al. 2016; Xu, Ding, et al. 2016).

Numerous studies have indicated that traditional Chinese medicine can play an important role in delaying aging and treating infertility (Zhu et al. 2017; Wen et al. 2018). Heshouwuyin is a Chinese herbal formula with *Polygonum multiflorum* (Thunb.) Haraldson (Polygonaceae) (dried root) [heshouwu] as the main medicine. We previously showed that Heshouwuyin has many curative effects (Niu et al. 2012, 2014; Chen et al. 2016). It can reduce the concentration of gonadotropin-releasing hormone (GnRH), luteinizing hormone (LH) and follicle-stimulating hormone (FSH) in aging rats and increase the testosterone concentration. It can also increase testicular indices, the number of seminiferous epithelia and the area of the seminiferous tubules in natural-aging rats; reduce cellular senescence and apoptosis rate in the testis tissue of natural-aging rats. In addition, it can reduce the expression of the senescence marker protein β-galactosidase in the testis tissue of aging rats and inhibit apoptosis; as well as improve the plasma membrane integrity, DNA integrity and mitochondrial function of sperm in naturally aging rats. Our previous gene chip results indicated that 912 differentially expressed genes are regulated by Heshouwuyin and the expression of several key genes in the insulin/IGF signalling pathway are altered. Therefore, we speculated that the insulin/IGF signalling pathway is involved in the Heshouwuyin-mediated regulation of testicular function in aging rats.

The insulin/IGF signalling pathway regulates a variety of cellular activities, including cell survival, proliferation, differentiation, and metabolism (Pitetti et al. 2013), and plays a crucial role in mammalian sexual development and testicular function by activating two related tyrosine kinase receptors, the INSR and IGF1R (Neirijnck, Papaioannou, et al. 2019). IGF1/IGF1R is involved in testicular development during embryonic development, promoting the proliferation of Sertoli and germ cells, as well as the differentiation of germ cells (Cannarella et al. 2018). The insulin/IGF signalling pathway in Leydig and Sertoli cells (SC) affects male reproductive function and regulates spermatogenesis (Cannarella et al. 2019; Neirijnck, Kuhne, et al. 2019; Neirijnck, Papaioannou, et al. 2019).

Using quantitative real-time polymerase chain reaction (qRT-PCR), immunofluorescence, western blotting, and flow cytometry, we observed the expression of INSR, IRS1, IRS2, IGF1, and IGFBP3 in the testicular tissue of naturally aging Wistar rats and aging spermatogenic cells. We also observed the activation of the insulin/IGF signalling pathway in spermatogenic cells after the administration of Heshouwuyin to further explore the molecular mechanism by which Heshouwuyin regulates the insulin/IGF signalling pathway.

## Materials and methods

### Animals and treatment

Specific pathogen free male Wistar rats 12 months old, weighing 320–360 g, were provided by the Experimental Animal Centre of Hebei Medical University (animal licence number: 1510063). The animal use protocol listed below was reviewed and approved by the Hebei University Animal Ethics and Welfare Committee (AEWC) and given the approval no. IACUC 2018018. Rats were housed in clean cages with a constant temperature (25 °C) and photoperiod (12 h light/dark cycle). All experimental procedures were conducted according to the guidelines of the Animal Care and Ethics Committee of Hebei University, China. The disposal of experimental animals was performed in accordance with the Guidance for the Care and Use of Laboratory Animals formulated by the Ministry of Science and Technology of China.

Heshouwuyin contains a mixture of *P. multiflorum* (dried root) [heshouwu], *Cistanche deserticola* Y.C. Ma (Orobanchaceae) [roucongrong], *Achyranthes bidentata* B1. (Amaranthaceae) (dried root) [niuxi], *Poria cocos* (Schw.) Wolf (Polyporaceae) (sclerotia) [fuling], *Epimedium brevicornu* Maxim. (Berberidaceae) [yinyanghuo], and *Salvia miltiorrhiza* Bge. (Labiatae) (dried root) [danshen] at a mass ratio of 3:2:3:2:5:3. The results of previous studies showed that the dose of 0.48 g (herb)/kg had the most significant protective effect on testicular function in aging rats (Wang et al. 2011). In this experiment, granules formulated by Guangdong Yifang Pharmaceutical Co., Ltd. were used. The equivalent ratios (mass ratio) of granules to herbs were as follows: *P. multiflorum* root 1:10, *C. deserticola*: 1:10, *A. bidentata* root: 1:5, *E. brevicornu*: 1:20, *S. miltiorrhiza* root: 1:10, and *P. cocos* sclerotia: 1:5. Based on the equivalent ratio of herbs to granules, the dose of the mixed granules administered to rats was 0.056 g/kg (obtained by dissolving 0.56 g of prepared Heshouwuyin granules in 0.8 mL of normal saline).

Twelve-month-old male Wistar rats (*n* = 30; 320–360 g) were randomly divided into a young control group, a natural-aging group, and a Heshouwuyin group (*n* = 10). The testes of the rats in the young control group were harvested after adaptive feeding for 1 week. Rats in the Heshouwuyin group and the natural-aging group were fed for 120 d to 16 months of age. The Heshouwuyin group was administered Heshouwuyin granules (0.056 g/kg) twice a day, and the natural-aging group was orally administered an equivalent volume of normal saline starting at 16 months of age for 60 d. At 18 months of age, the left testes of the rats in the Heshouwuyin group and the natural-aging group were removed under anaesthesia using sodium pentobarbital (50 mg/kg). At the same time, the right testicular tissue was stored at −80 °C for subsequent use.

### Preparation of serum

Twelve-month-old male Wistar rats weighing 320–360 g (*n* = 20) were orally administered Heshouwuyin granules (prepared as described above) twice a day for 7 consecutive days to obtain Heshouwuyin-containing serum and were administered the same amount of normal saline to obtain blank serum. On day 7, the rats were anaesthetised with sodium pentobarbital (50 mg/kg) 1 h after drug administration, and blood was withdrawn aseptically from the abdominal aorta. The serum was separated, inactivated at 56 °C for 30 min, filtered through 0.22-μm filters, aseptically aliquoted, and stored at −80 °C.

### Cell culture and identification

On day 6 after birth, the spermatogenic epithelium of male rats contains only original type A spermatogonia and a small number of SC. B-type spermatogonia appear on day 8. Meiosis prophase occurs at 10–14 d, at which time SC and A- and B-type spermatogonia rapidly proliferate. The abundance of SC increases on days 14–20 (Cao et al. 2014). Germ cells reach the early and late thick-line stages on day 14 and 18, respectively, and a large number of secondary spermatocytes and haploid sperm cells appear on days 18–20 (Paolo et al. 2016). Therefore, spermatogonial stem cells (SSCs) were isolated from 7- to 9-day-old Wistar rats for culture, whereas Sertoli and Leydig cells were isolated from 15- to 20-day-old Wistar rats for culture.

#### Sertoli cells

**Figure 1.**
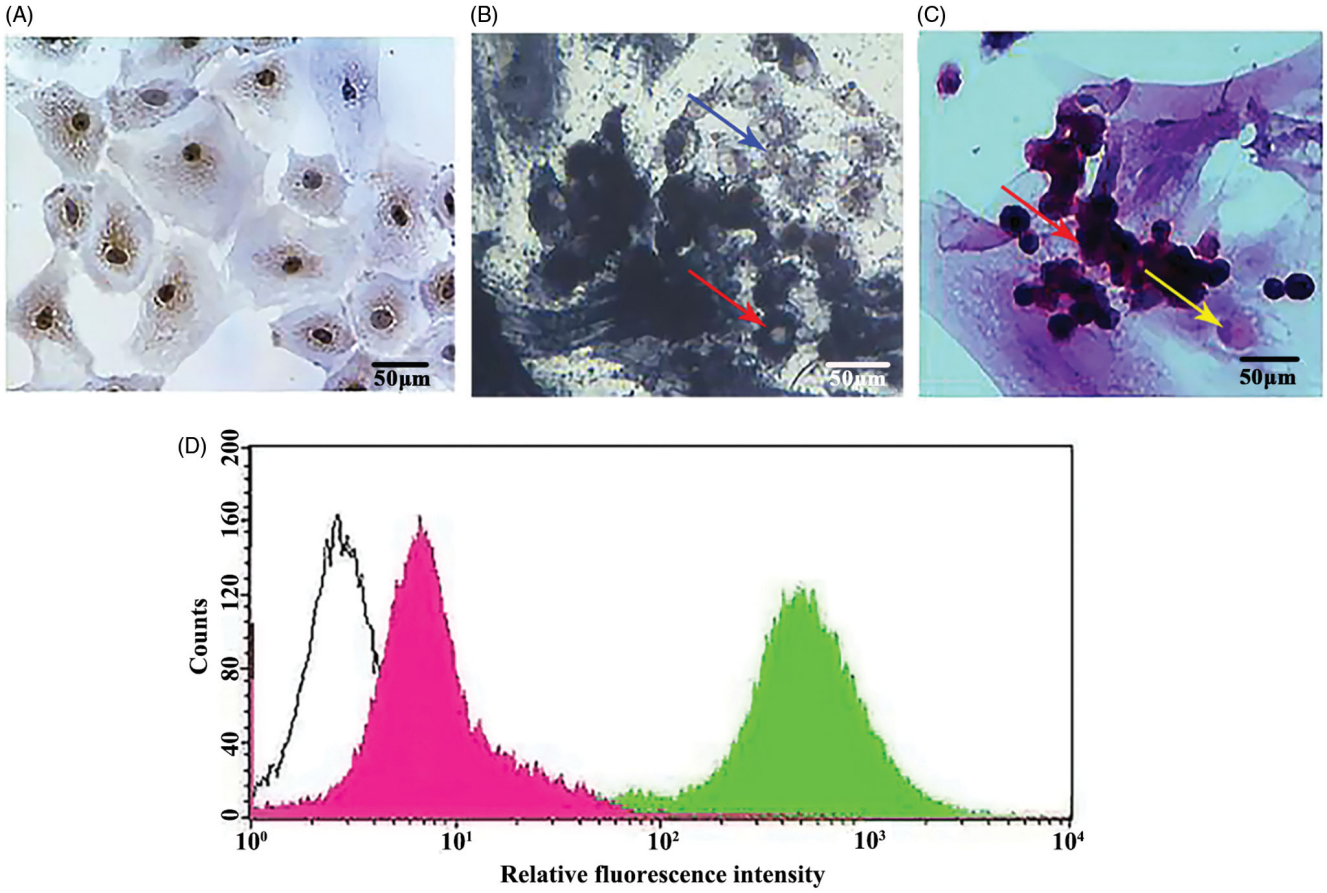
Identification of Sertoli cells, spermatogenic cells, and SSCs. (A) Sudan IV staining of Sertoli cells; original magnification, ×400. (B) Alkaline phosphatase staining showing spermatogonial cell membrane/cytoplasm staining with grey–black particles (indicated by the red arrow) and sperm cells as weakly positive/not coloured (indicated by the green arrow); original magnification, ×400. (C) Haematoxylin and eosin (H&E) staining of spermatogenic cells (spermatogonial cells indicated by the red arrow and Sertoli cells indicated by the yellow arrow); original magnification, ×400. (D) Flow chart of SSCs (the white, pink, and green peaks represent negative control, α6-integrin+, and β1-integrin+ cells, respectively).

Because SC provide growth factors and nutrients for germ cells, this experiment used spermatogenic cells and SC for coculture (Griswold 2018). The testicular tissue of Wistar rats was harvested on days 15–20 after birth. Fat pads and fascia were separated to maintain the integrity of the testis. The tissue was digested with type I collagenase to open the seminiferous tubules and free the Leydig cells. The Leydig cells were allowed to stand and the adherent cells attached to the plate. The spermatogenic tubules were dissociated by trypsin digestion and then cultured in DMEM/F12 medium containing 10% FBS in a 35 °C incubator for 4 h. After the Leydig cells and fibroblasts present in the cell suspension attached, the remaining suspended cells were plated in a new 6-well cell culture plate at a density of 2 × 10^5^ cells per well. After 3 d of culture, the SC were identified by Sudan IV staining (Figure 1(A)). The cytoplasm showed the enrichment of orange–red lipid droplets, which accumulated at the cytoplasmic poles or were dispersed around the nucleus. The cell purity was assessed to be greater than 90%.

#### Spermatogonial stem cells

SSCs were isolated using type IV collagenase (Sigma, St. Louis, MO) and trypsin digestion from the testis tissue of 7- to 9-day-old Wistar rats. The stem cells were isolated and purified by a two-step enzymatic digestion and differential adherence (Liu S et al. 2011). SSCs were cultured in DMEM/F12 medium containing 15% FBS in a 35 °C incubator for 3 h. After most of the Leydig cells and fibroblasts had attached, the supernatant was transferred, and the cells were counted. Sertoli cells were inoculated with SSCs at 3–5 × 10^5^ cells/mL and cultured for 3 d. SSCs were identified via the detection of SSC-specific surface receptors, α6-integrin and β1-integrin, by flow cytometry (BD FACSCalibur, Bergen County, NJ, USA) using fluorescein isothiocyanate-labeled α6-integrin and β1-integrin antibodies (BD Company, USA). The results showed that the expression rates of the negative control, α6-integrin+, and β1-integrin + cells were 10.71, 26.76, and 99.13%, respectively. Compared with that in the negative control, the fluorescence intensity of α6-integrin + and β1-integrin + cells was higher, and the peaks shifted to higher values (Figure 1(D)).

#### Spermatogenic cells

SSCs (3–5 × 10^5^ cells/mL) were inoculated with SC that had been cultured for 72 h. Then, all cells were cultured in DMEM/F12 medium containing 15% FBS for 7 d (He et al. 2015) and were identified using alkaline phosphatase staining, which stains spermatogonia blue–black. Sertoli cells did not take up any of the alkaline phosphatase stain, whereas spermatocytes, sperm cells, and sperm showed only scant levels of staining (Figure 1(B)). Haematoxylin and eosin staining indicated that spermatogenic cells had a round morphology, formed clusters and were stained purple, whereas the SC were stained lavender and showed irregular morphological shapes (Figure 1(C)).

### Cell experiment

#### Establishment of a spermatogenic cell aging model

**Figure 2.**
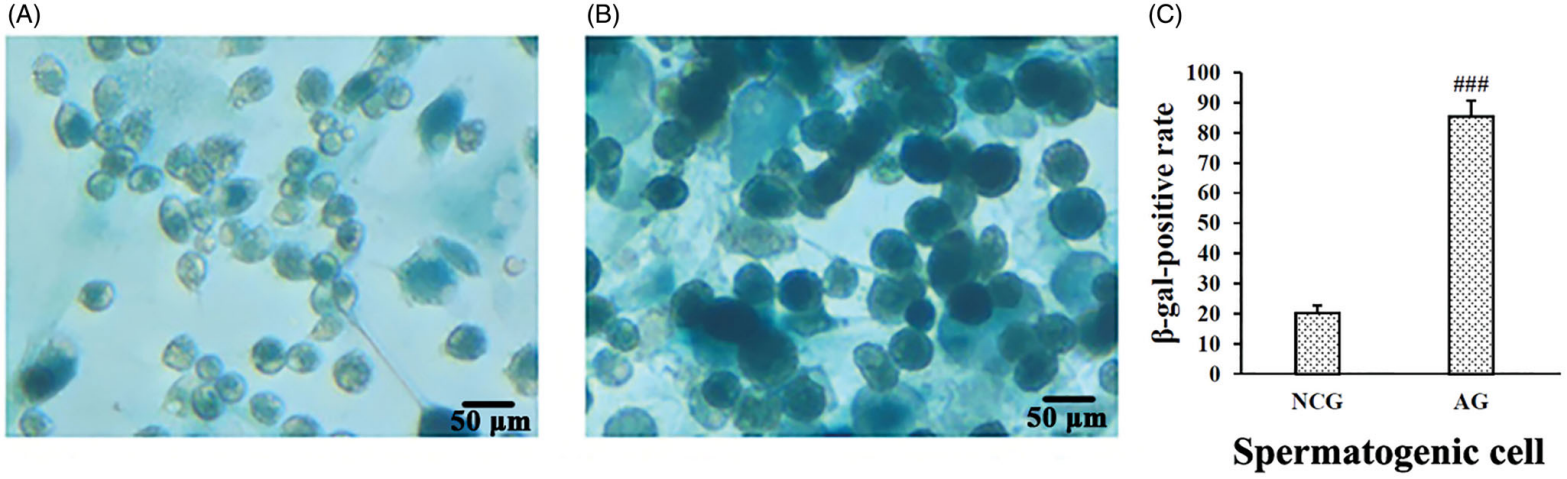
Results of the β-galactosidase staining in the spermatogenic cell aging model. β-Galactosidase staining was performed using (A) the normal control group (NCG; original magnification, ×400) and (B) the aging group (AG; original magnification, ×400). (C) Quantitative analysis of the positive β-galactosidase staining in spermatogenic cells. Data are expressed as the mean ± S.D. ^###^*p* < 0.001 vs. the NCG in spermatogenic cells (*n =* 3).

Senescence-associated β-galactosidase (SA-β-gal) is the most widely used biomarker for senescent cells (Hall et al. 2017). Cells were stained with a β-galactosidase staining kit (Beyotime, Shanghai, China). Cultured SSCs were cocultured with SC for 7 d in DMEM/F12 medium containing 15% FBS and then incubated with final concentrations of 50 μmol/L H_2_O_2_ and 100 μmol/L FeSO_4_ for 8 h to induce spermatogenesis (Shan et al. 2019). The supernatant was discarded, the cells were washed once with phosphate-buffered saline (PBS), and the medium was replaced with fresh culture medium. The cells were cultured for another 72 h and then β-galactosidase staining was repeated three times; the number of blue-stained cells per 500 cells was recorded. More than 80% of the spermatogenic cells were β-galactosidase positive (Figure 2(A–C)).

#### Determination of optimal concentrations of active compounds

**Figure 3.**
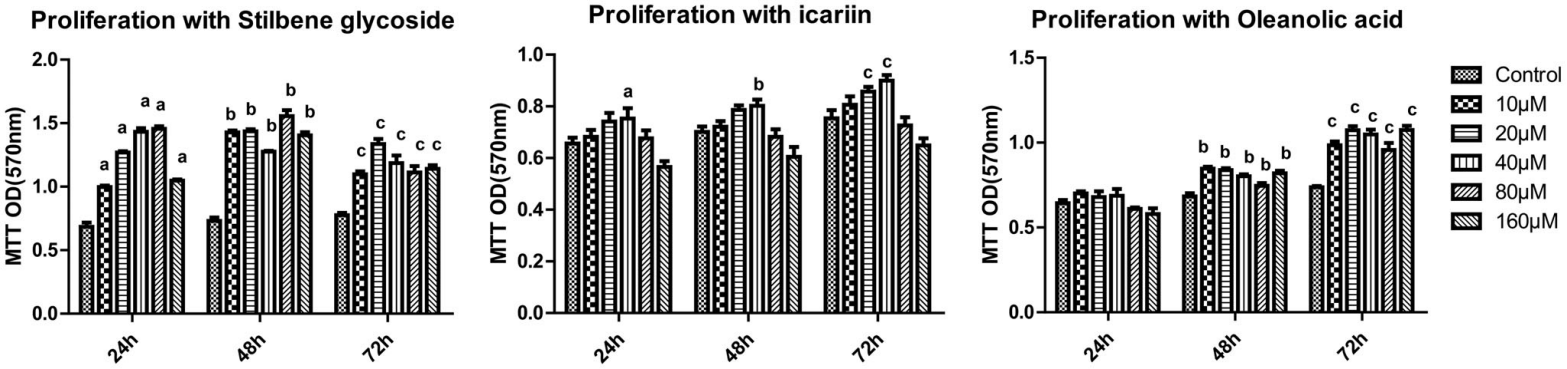
Effects of stilbene glycoside, icariin, and oleanolic acid on the viability of spermatogenic cells. ^a^*p* < 0.05 vs. 24 h control, ^b^*p* < 0.05 vs. 48 h control, ^c^*p* < 0.05 vs. 72 h control (*n* = 3).

The 3-(4,5-dimethylthiazol-2-yl)-2,5-diphenyltetrazolium bromide (MTT) method was used to screen for the optimal concentrations of icariin (active compound in *E. brevicornu*), oleanolic acid (active compound in *C. deserticola*), and stilbene glycoside (active compound in *P. multiflorum* root) to be used for the treatment of spermatogenic cells. The experiments were performed using an MTT kit according to the manufacturer’s protocol (Beyotime, Shanghai, China). Sertoli cells were transferred to 96-well cell culture plates at a density of 2 × 10^3^. After 72 h of culture, the SSCs were seeded into each well at a density of 5 × 10^3^. After 7 d of culture in DMEM/F12 medium containing 15% FBS, icariin, oleanolic acid and stilbene glycoside (Chengdu Phytochemical Pure Biotechnology Co., Ltd., Chengdu, China) were added to the culture medium at final concentrations of 0 (control), 10, 20, 40, 80, and 160 μM. At 24, 48 and 72 h, 10 μL of MTT solution (5 mg/mL) was added to each well and the plate was incubated at 37 °C in a 5% CO_2_ incubator for 4 h. Then, 100 μL of formazan solvent was added to each well, and after 3 h of incubation, the absorbance of each well was measured at 570 nm using a microplate reader (Biotek Epoch, Winooski, VT, USA). Five replicate wells were established for each group, and three independent experiments were repeated. The optimal concentrations were 40 μM, 40 μM, and 50 μM for icariin, oleanolic acid, and stilbene glycoside, respectively, and the duration of treatment was 72 h (Figure 3).

#### Cell grouping and treatment

For this experiment, cell cultures were divided into a normal control group, an aging group, a Heshouwuyin group, a stilbene group, an icariin group, and an oleanolic acid group. The normal control group of spermatogenic cells was cultured for 10 d. The other groups were treated as follows. Spermatogenic cells were cultured in DMEM/F12 medium containing 15% FBS for 7 d, followed by the addition of 50 μmol/L H_2_O_2_ and 100 μmol/L FeSO_4_, which were removed after 8 h of incubation. The cells in the aging group were cultured in DMEM/F12 medium containing 5% FBS and 10% blank rat serum for 72 h. The Heshouwuyin group was treated with 10% Heshouwuyin-containing serum in DMEM/F12 medium containing 5% FBS for 72 h. The other treatment groups were treated with 1 μL DMSO containing stilbene glycoside, icariin, or oleanolic acid in DMEM/F12 medium with 5% FBS and 10% blank rat serum for 72 h (the normal control, aging and Heshouwuyin groups were treated with 1 μL of DMSO).

### Inhibitor concentration screening and cell grouping

**Figure 4.**
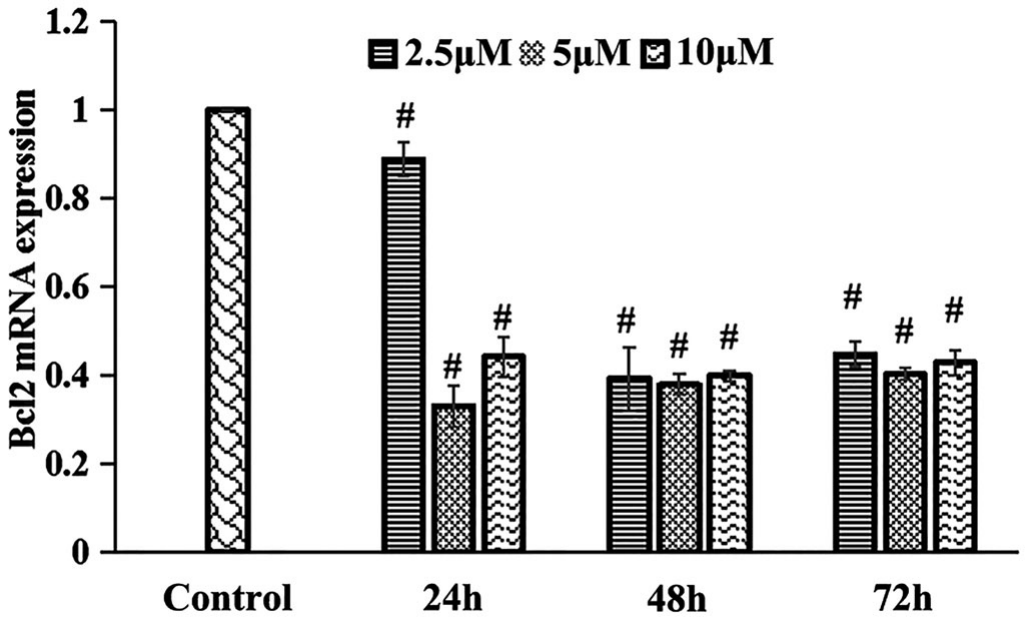
qRT-PCR was used to detect the mRNA expression of BCL-2, a target gene downstream of the insulin/IGF signalling pathway, and to determine the optimal concentration and duration of action of the inhibitor. #*p* < 0.01 vs. control (*n* = 3).

The IGF-1 receptor/InsR inhibitor BMS-754807 (MedChemExpress, Monmouth Junction, USA) was dissolved in DMSO. BMS-754807 was added to the spermatogenic cells cultured for 7 d at a final concentration of 2.5, 5, and 10 μM, and each concentration was applied for 24, 48 and 72 h, respectively. The expression of BCL-2, which is a downstream target gene of the insulin/IGF signalling pathway, was detected by qRT-PCR, and the optimal concentration and duration of the inhibitor treatment were determined to be 5 μM for 24 h (Figure 4). After 7 d of spermatogenic cell culture, the cells were divided into the normal group, the inhibitor group and the Heshouwuyin group. The normal group and the inhibitor group were treated with 1 μL of DMSO or 1 μL of BMS-754807 in DMSO solution in DMEM/F12 containing 15% FBS. After 24 h, the solution was changed, 5% FBS and 10% blank rat serum were added to the DMEM/F12 and the cells were cultured for 72 h. In the Heshouwuyin group, 1 μL of BMS-754807 in DMSO was added to DMEM/F12 containing 15% FBS. The culture was continued for 24 h. After 24 h, the medium was changed. DMEM/F12 containing 5% FBS and 10% Heshouwuyin-containing serum was added to the culture, which was then incubated for 72 h. qRT-PCR was used to measure the expression of the BCL-2 gene in each group.

### Cell cycle

Cell cycle analysis was performed using a flow cytometer (BD FACSCalibur, Bergen County, NJ). The cells from the normal group, the inhibitor group and the Heshouwuyin group were trypsinized into suspension. Subsequently, the cells were thoroughly suspended (∼1.0 × 10^6^) in 1 mL of PBS and fixed overnight at 4 °C with 70% ethanol. The fixed cells were washed and suspended in a mixed solution of propidium iodide (PI, 50 μg/mL) and RNase (100 μg/mL; both from Beyotime, Shanghai, China) for 30 min, and the cell cycle was analyzed by flow cytometry.

### Immunofluorescence staining

#### Testicular tissue

Tissue sections were obtained from one testicle of three rats in each group; five sections of testicular tissue were selected for immunolabeling. Cryosections (8 μm thick) of testicular tissue were washed with PBS and permeabilized with 0.5% Triton X-100 (Sigma, St. Louis, MO) for 10 min. Non-specific sites were blocked by incubation with 5% serum albumin for 3 h at 25 °C, and the sections were incubated with primary antibodies against INSR, IRS1, IRS2, IGF1, or IGFBP3 (all diluted 1:100; Santa Cruz Biotechnology, Dallas, TX) at 4 °C overnight. Then, the sections were washed with PBS and incubated with a secondary antibody (1:2000; Proteintech, Hubei, China) for 1 h at 37 °C. Subsequently, the sections were stained with 4′,6-diamidino-2-phenylindole (DAPI; Sigma) for 20 min at room temperature, washed with PBS, dried at room temperature, and sealed with 50% glycerol. To obtain the rate of positive cells, the number of positive cells and the total number of cells were counted in each of 10 randomly selected fields of view under a fluorescence microscope (Electron Microscopy Sciences, Hatfield, PA). Sections of the corresponding parts of the testis of one of the three rats in each group were sectioned. Five sections of testicular tissue were selected for staining.

#### Spermatogenic cells

The cells were trypsinized and transferred to six-well plates. Each group of cells was washed with PBS, fixed with 4% paraformaldehyde for 30 min, permeabilized with 0.5% Triton X-100 (Sigma) for 20 min, and again washed with PBS. Non-specific sites were blocked by incubation with 5% serum albumin for 20 min at room temperature, and then, the cells were incubated with primary antibodies against INSR, IRS1, IRS2, IGF1, and IGFBP3 (all diluted 1:100; Santa Cruz Biotechnology) overnight at 4 °C. The cells were washed with PBS and incubated with a secondary antibody (1:2000; Proteintech) for 1 h at 37 °C. The cells were then incubated with DAPI (Sigma) for 20 min at room temperature, washed with PBS, dried at room temperature, and sealed with 50% glycerol. The number of positive cells was obtained for each of 10 randomly selected fields of view under a fluorescence microscope (Electron Microscopy Sciences). Each experiment was repeated three times.

### RNA isolation and qRT-PCR

qRT-PCR was used to assess the mRNA expression levels of INSR, IRS1, IRS2, IGF1, and IGFBP3, the key genes in the insulin/IGF signalling pathway of testicular tissue and spermatogenic cells, and the mRNA expression level of BCL-2, a downstream target gene of the insulin/IGF signalling pathway in spermatogenic cells. Total RNA was extracted from tissues and cells using TRIzol reagent (Ambion, Austin, TX), and the mRNA was reverse-transcribed to cDNA using a reverse transcription kit (TaKaRa, Tokyo, Japan) as per the manufacturer’s instructions. cDNA was used as a template for qRT-PCR analysis, and qRT-PCR amplification was performed using SYBR Green PCR master mix (TaKaRa) per the manufacturer’s instructions. The results were analyzed using the 2^−ΔΔCt^ method. The primer sequences used for qRT-PCR are shown in Table 1.

**Table 1.** qPCR primer sequence.

Gene	Primer sequences	Annealing Tm (°C)	Product size (bp)
INSR	F: GGCCCGATGCTGAGAACA	59 °C	188
	R: CGTCATTCCAAAGTCTCCGA		
IRS1	F:GTTTCCAGAAGCAGCCAGAG	60 °C	474
	R:ACTCTCTCCACCCAACGTGA		
IRS2	F:TGCGAACAGCCGTCGGTGAC	65 °C	103
	R:GACCGGTGACGGCTGAACGG		
IGF-1	F:ACGCTGTCTACCAGATTCCC	64 °C	98
	R:TGCCCACAAACTCCTCAAAC		
IGFBP3	F:GTCCAAGCGGGAGACAGAATA	60 °C	313
	R:TGAACCCGTCATACGTCCAT		
BCL2	F:GAGGATTGTGGCCTTCTTTG	56 °C	108
	R:AGGTACTCAGTCATCCACA		
β-actin	F:TCACCCACACTGTGCCCATCTACGA	58 °C	348
	R:GGATGCCACAGGATTCCATACCCA		

### Western blot analysis

Tissue or cells were lysed in RIPA buffer with phenylmethylsulfonyl fluoride (99:1; Beyotime, Shanghai, China). The protein concentration was measured using a Bicinchoninic Acid Assay kit (Thermo Scientific, Boca Raton, FL; Pierce is one of the product lines of Thermo Scientific) as per the manufacturer’s instructions. Then, 60 μg of protein was separated by sodium dodecyl sulfate-polyacrylamide gel electrophoresis, and the separated proteins were transferred to a polyvinylidene difluoride membrane (Millipore, Atlanta, GA). After being blocked with 5% skim milk at room temperature for 1 h, the membrane was incubated with primary antibodies against INSR, IRS1, IRS2, IGF1, and IGFBP3 (all diluted 1:300; Santa Cruz Biotechnology) at 4 °C overnight. The membrane was then incubated with the respective horseradish peroxidase-conjugated secondary antibodies (1:5000; Proteintech) at room temperature for 1 h and washed five times with PBS containing Tween 20 for 5 min per wash. Finally, the membranes were developed using a Pierce enhanced chemiluminescence system (Thermo Scientific, Boca Raton, FL). This experiment was repeated three times, and the grey value of each protein in the obtained images was analyzed using ImageJ software. β-Actin served as the loading control.

### Statistical analysis

The experimental data (x±s) were analyzed by SPSS 19.0 statistical software. First, a normality test was performed. One-way ANOVA was used for the analysis of data that satisfied the normal distribution criteria. If the homogeneity of the variance was satisfied, the least significant difference (LSD) was used for the pairwise comparison, and Dunnett's T3 method was used if the variance was not uniform.

## Results

### Effects of heshouwuyin on INSR, IRS1, IRS2, IGF1 and IGFBP3 genes in testis

**Figure 5.**
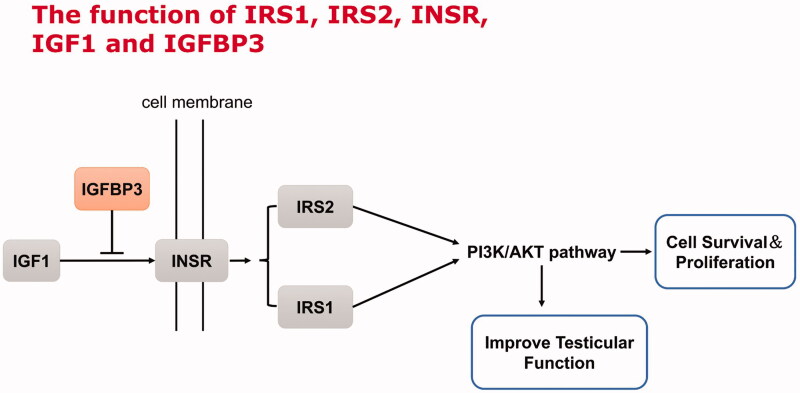
The function of IRS1, IRS2, INSR, IGF1 and IGFBP3.

**Figure 6.**
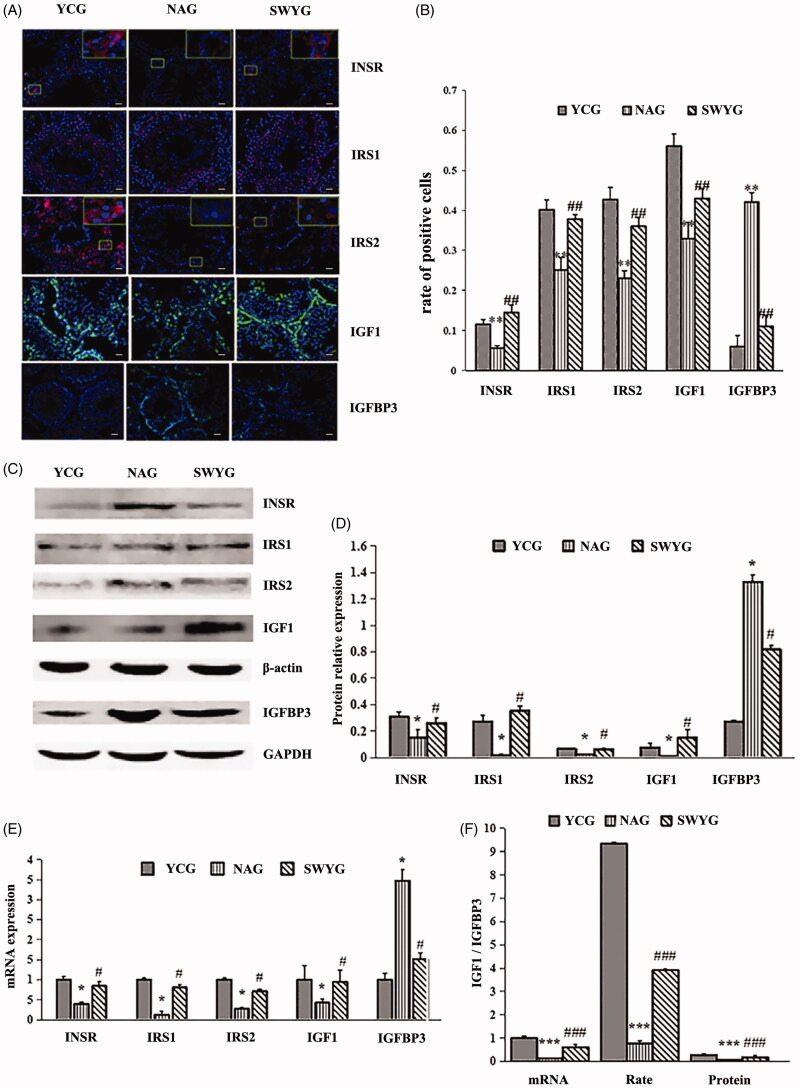
Effect of Heshouwuyin on the expression of insulin/IGF signalling pathway-related genes in the testes. The mRNA and protein expression of INSR, IRS1, IRS2, IGF1, and IGFBP3 in the testes of Wistar rats in the control group, natural-aging group, and Heshouwuyin-treated group as detected by RT-PCR, Western blotting, and immunofluorescence. (A) Representative images of INSR, IRS1, IRS2, IGF1, and IGFBP3 expression in the young control group, natural-aging group, and Heshouwuyin-treated group; original magnification, ×400 (scale bar: 20 μm). (B) Determination of the positive INSR, IRS1, IRS2, IGF1, and IGFBP3 expression rate in the young control group, natural-aging group, and Heshouwuyin-treated group. (C, D) Protein expression of INSR, IRS1, IRS2, IGF1, and IGFBP3 in the young control group, natural-aging group, and Heshouwuyin-treated group. (E) RT-PCR was used to detect the mRNA expression of INSR, IRS1, IRS2, IGF1, and IGFBP3 in the young control group, natural-aging group, and Heshouwuyin-treated group. (F) Quantitative analysis of the mRNA and protein expression and IGF1/IGFBP3-positive cell rate in the young control group, natural-aging group, and Heshouwuyin-treated group. 1. NAG; 2. YCG; 3. SWYG. YCG: young control group; NAG: natural-aging group; SWYG: Heshouwuyin group. Data are expressed as the mean ± S.D. *p < 0.05, **p < 0.01, ***p < 0.001 vs. YCG; #*p* < 0.05, ##*p* < 0.01, ###*p* < 0.001 vs. NAG (*n* = 3).

IGF1, INSR, IRS1, IRS2, and IGFBP3 are the key factors of the insulin/IGF signalling pathway. Activation of the insulin/IGF signalling pathway activates the PI3K/AKT pathway, which in turn promotes proliferation and differentiation of germ cells and testicular function (Cannarella et al. 2018) (Figure 5). We used qRT-PCR, immunofluorescence, and Western blotting to evaluate the expression of key genes and proteins in the testes. Immunofluorescence labelling showed positive expression of INSR, IRS1, IRS2, and IGF1 as red granules, while that of IGFBP3 was indicated by a green signal. Normal nuclei were stained blue with DAPI. Immunofluorescence staining showed that the INSR-positive red signal was distributed throughout the cytomembrane, while the IRS1-, IRS2-, and IGF1-positive granules and the IGFBP3-positive green signal were mainly located in the cytoplasm. INSR was mainly expressed in Leydig cells, myoid cells, and several spermatogonia. IRS1 was mainly expressed in SC, myoid cells, and spermatocytes. IRS2 was mainly expressed in Leydig cells, myoid cells, SC, and spermatocytes. IGF1 was mainly expressed in spermatids and, at low levels, in spermatogonia and Leydig cells. IGFBP3 was mainly expressed in spermatogonia and spermatocytes (Figure 6(A)). These findings show that the expression of INSR, IRS1, IRS2, and IGF1 was lower in the natural-aging group than the young control group, whereas IGFBP3 expression was higher in the natural-aging group. The expression of INSR, IRS1, IRS2, and IGF1 was significantly higher and that of IGFBP3 was lower in the Heshouwuyin-treated group compared to the aging group (Figure 6(B), Table 2).

**Table 2.** Positive cell rate of INSR, IRS1, IRS2, IGF1, and IGFBP3 expression in each group.

	YCG	NAG	SWYG
INSR	0.116 ± 0.011	0.056 ± 0.006*	0.144 ± 0.019^†^
IRS1	0.401 ± 0.026	0.251 ± 0.031*	0.378 ± 0.011^†^
IRS2	0.427 ± 0.031	0.230 ± 0.019*	0.360 ± 0.022^†^
IGF1	0.560 ± 0.031	0.330 ± 0.040*	0.430 ± 0.024^†^
IGFBP3	0.060 ± 0.027	0.420 ± 0.024*	0.110 ± 0.028^†^

YCG: young control group; NAG: natural-aging group; SWYG: Heshouwuyin group.

Data are expressed as mean ± S.D.

*p < 0.01 vs. YCG;

^†^*p* < 0.01 vs. NAG (*n* = 3).

Semi-quantitative statistical analysis of the Western blots showed that the trend of variation in the expression of INSR, IRS1, IRS2, IGF1, and IGFBP3 was consistent with that obtained using immunofluorescence (Figure 6(C,D)). The variation trend in the mRNA expression levels was consistent with that of the protein expression (Figure 6(E)). These results are consistent with the results of the microarray analysis.

A decrease in the apoptosis of testicular tissues and spermatogenic manifests as an increase in the IGF1/IGFBP3 ratio (Lue et al. 2010). Therefore, we determined the IGF1/IGFBP3 ratio in the testicular tissue and found that the ratio was significantly decreased in the aging group compared with the young control group at both the mRNA and protein levels. However, the IGF1/IGFBP3 ratio was significantly higher in the Heshouwuyin-treated group than the aging group (Figure 6(F)).

Our research showed that Heshouwuyin can regulate the gene expression of INSR, IRS1, IRS2, IGF1, and IGFBP3 in testicular tissue.

### Regulatory effect of Heshouwuyin on the genes of spermatogenic cells INSR, IRS1, IRS2, IGF1, and IGFBP3

#### Immunofluorescence staining results

**Figure 7.**
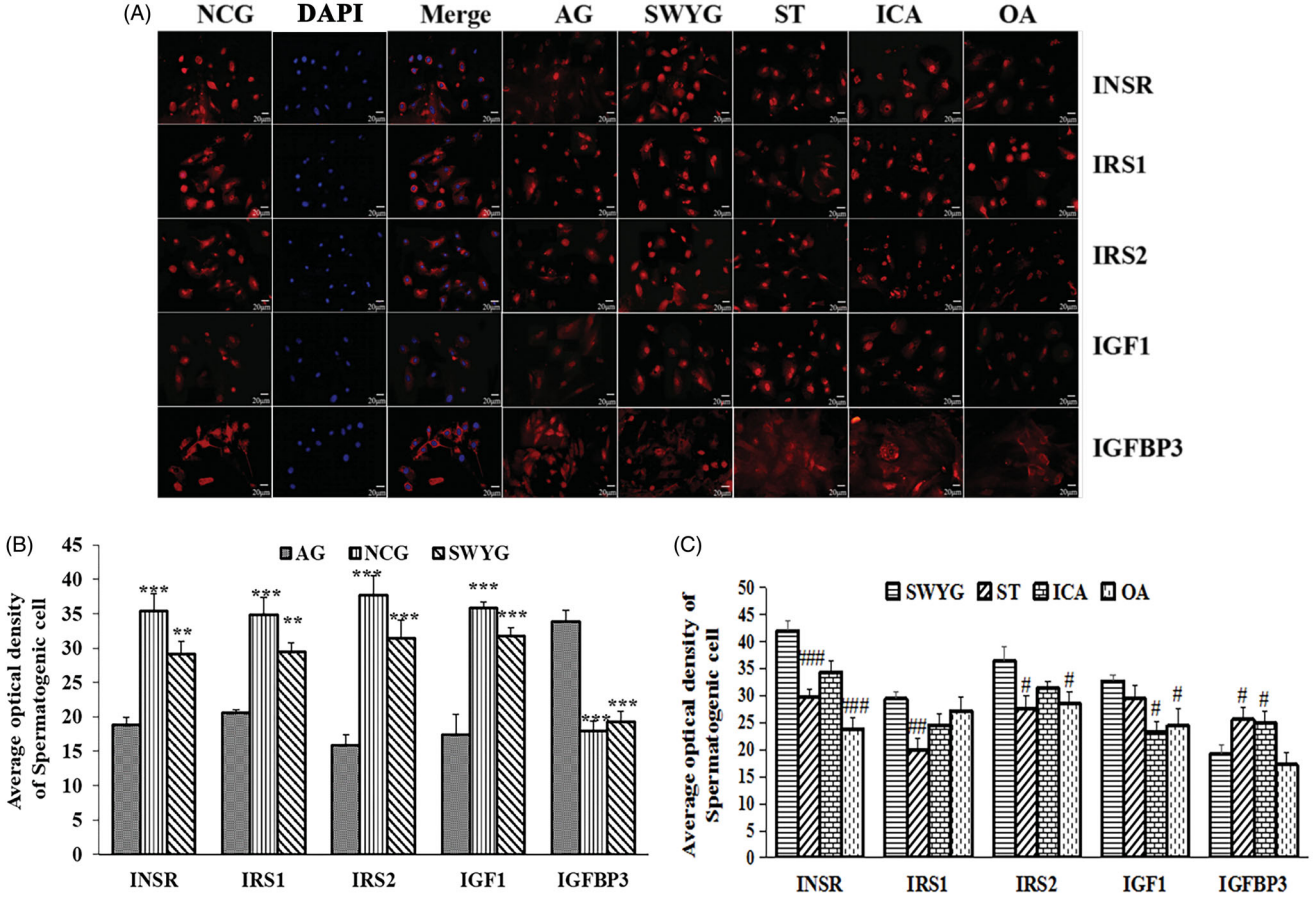
Results of the fluorescent labelling of key proteins in the insulin/IGF signalling pathway in spermatogenic cells in each group. (A) Immunoblotting analysis of spermatogenic cells from each group was conducted using primary antibodies specific for INSR, IRS1, IRS2, IGF1, and IGFBP3; original magnification, ×400 (scale bar: 20 μm). (B) Quantitative analysis of the expression of INSR, IRS1, IRS2, IGF1, and IGFBP3 in the normal group, natural-aging group, and Heshouwuyin-treated group. (C) Quantitative analysis of the expression of INSR, IRS1, IRS2, IGF1, and IGFBP3 in the stilbene-treated group, icariin-treated group, and oleanolic acid-treated group. 1: NCG; 2: AG; 3: SWYG; 4: ST; 5: ICA; 6: OA. NCG: normal control group; AG: natural-aging group; SWYG: Heshouwuyin-treated group; ST: stilbene; ICA: icariin; OA: oleanolic acid. Data are expressed as the mean ± S.D. **p < 0.01, ***p < 0.001 vs. AG; #*p* < 0.05, ###*p* < 0.001 vs. the SWYG (*n* = 3).

**Figure 8.**
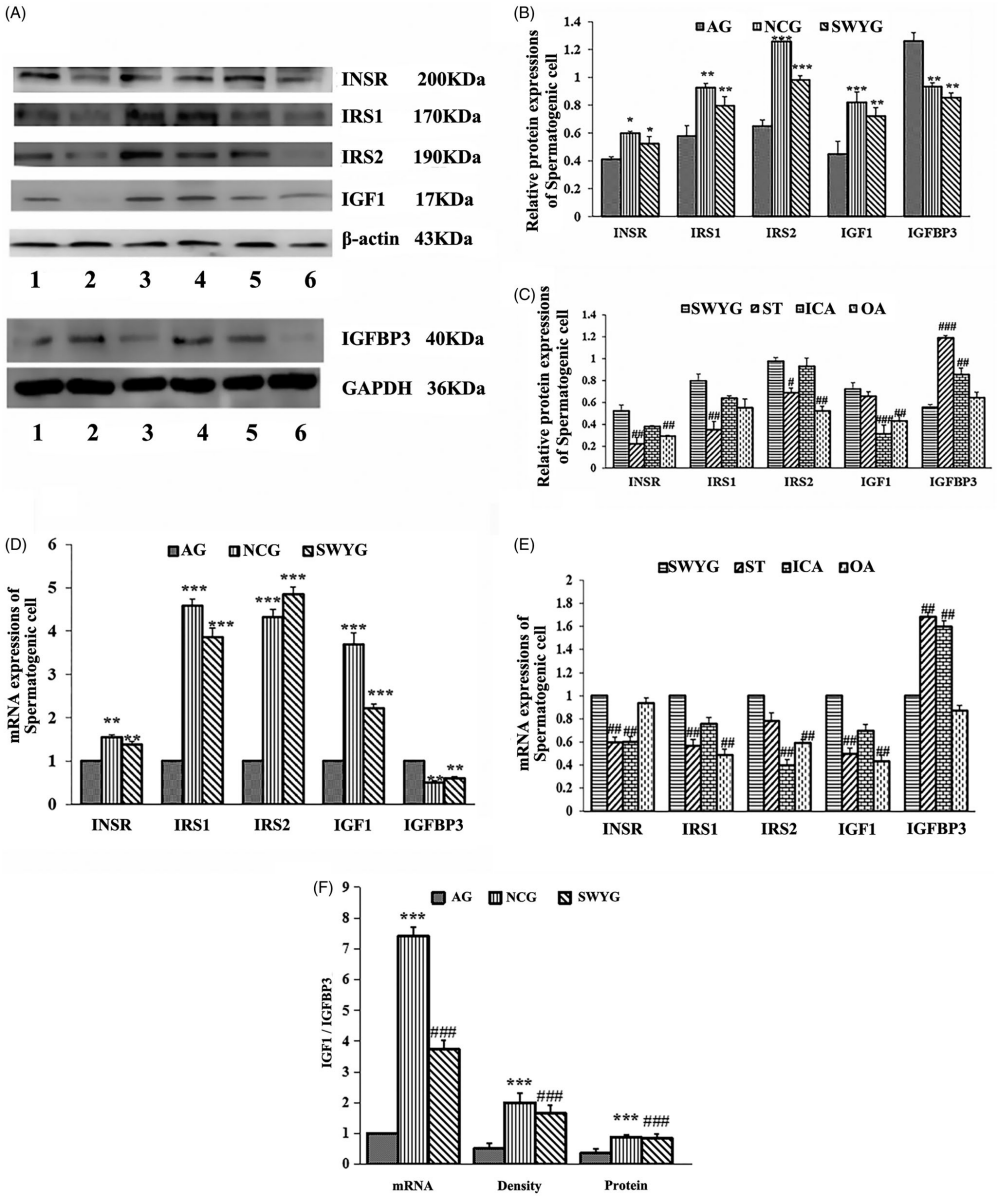
Effect of Heshouwuyin on the expression of insulin/IGF signalling pathway-related genes in spermatogenic cells. (A) Protein expression of INSR, IRS1, IRS2, IGF1, and IGFBP3 in different groups. (B) Quantitative analysis of the expression of INSR, IRS1, IRS2, IGF1, and IGFBP3 in the normal group, the aging model group, and the Heshouwuyin-treated group. (C) Quantitative analysis of INSR, IRS1, IRS2, IGF1, and IGFBP3 protein expression in the Heshouwuyin-treated group, stilbene glycoside-treated group, icariin-treated group, and oleanolic acid-treated group. (D) qRT-PCR analysis of the expression of INSR, IRS1, IRS2, IGF1, and IGFBP3 in the normal group, the aging model group, and the Heshouwuyin-treated group. (E) qRT-PCR analysis of the expression of INSR, IRS1, IRS2, IGF1, and IGFBP3 in the Heshouwuyin-treated group, stilbene glycoside-treated group, icariin-treated group, and oleanolic acid-treated group. (F) Quantitative analysis of the ratio of IGF1/IGFBP3 mRNA, protein expression and average optical density in the normal group, aging model group and Heshouwuyin-treated group. 1: NCG; 2: AG; 3: SWYG; 4: ST; 5: ICA; 6: OA. NCG: normal control group; AG: aging group; SWYG: Heshouwuyin-treated group; ST: stilbene; ICA: icariin; OA: oleanolic acid. Data are expressed as the mean ± S.D. *p < 0.05, **p < 0.01, ***p < 0.001 vs. AG; #*p* < 0.05, ##*p* < 0.01, ###*p* < 0.001 vs. the SWYG (*n* = 3).

The nuclear DAPI staining is shown in blue, and the INSR-, IRS1-, IRS2-, IGF1-, and IGFBP3-positive staining is shown as red fluorescence (Figure 7(A)). The average optical density of INSR, IRS1, IRS2, and IGF1 in the aging group was significantly lower than that in the normal control group, whereas the IGFBP3 average optical density was higher in the aging group than the normal control group. The average optical density of the INSR-, IRS1-, IRS2-, and IGF1-positive cells in the Heshouwuyin-treated group was significantly higher than that in the aging group, and the average optical density of IGFBP3-positive cells was significantly lower than that in the aging group (Figure 7(B)). The average optical density of INSR- and IRS2-positive cells in the Heshouwuyin-treated group was higher than that in the oleanolic acid- and stilbene glycoside-treated groups; however, there was no significant difference between the Heshouwuyin- and icariin-treated groups. The average optical density of IGF1-positive cells in the Heshouwuyin-treated group was significantly higher than that in the icariin- and oleanolic acid-treated groups; however, there was no significant difference between the Heshouwuyin- and stilbene glycoside-treated groups. The average optical density of IRS1-positive cells in the Heshouwuyin-treated group was higher than that in the stilbene glycoside-treated group; however, there were no significant differences between the Heshouwuyin-treated group and other treatment groups. The average optical density of IGFBP3-positive cells in the Heshouwuyin-treated group was lower than that in the stilbene glycoside- and oleanolic acid-treated groups; there was no significant difference between the stilbene glycoside- and oleanolic acid-treated groups. Overall, this analysis shows that the expression in the Heshouwuyin-treated group is greater than that in the other groups (Figure 7(C)). Semi-quantitative analysis by Western blot showed that the expression levels of INSR, IRS1, IRS2, IGF1, and IGFBP3 in each group were consistent with those observed by fluorescent immunostaining (Figure 8(A–C)).

The results obtained using qRT-PCR indicated that the expression levels of INSR, IRS1, IRS2, IGF1, and IGFBP3 were significantly lower and the expression of IGFBP3 was significantly higher in the aging group compared to the normal control group. After treatment with Heshouwuyin, the expression of INSR, IRS1, IRS2, and IGF1 in spermatogenic cells of the Heshouwuyin-treated group was significantly higher than that in the aging group. The expression of IGFBP3 was lower in the spermatogenic cells of the Heshouwuyin-treated group than the aging group (Figure 8(D)). Semi-quantitative analysis of the qRT-PCR data showed that the trend for the expression of INSR, IRS1, IRS2, IGF1, and IGFBP3 in spermatogenic cells was consistent with that in the testicular tissue. The mRNA expression of INSR, IRS1, and IGF1 in the Heshouwuyin-treated group was higher than that in the stilbene glycoside- and icariin-treated groups, whereas there was no significant difference between the Heshouwuyin- and oleanolic acid-treated groups. The mRNA expression of IRS2 was higher in the oleanolic acid- and icariin-treated groups than the stilbene glycoside- and Heshouwuyin-treated groups. The expression level of IGFBP3 mRNA in the Heshouwuyin-treated group was lower than that in the stilbene glycoside- and icariin-treated groups, but there was no significant difference from that in the oleanolic acid-treated group (Figure 8(E)). These results indicate that Heshouwuyin exerts stronger effects than the other test compounds. We determined the IGF1/IGFBP3 ratio in spermatogenic cells and found that the ratio was significantly lower in the aging group than the normal group at both the mRNA and protein levels. However, the IGF1/IGFBP3 ratio was significantly higher in the Heshouwuyin-treated group than the aging group (Figure 8(F)).

Our research showed that Heshouwuyin can regulate the expression of INSR, IRS1, IRS2, IGF1, and IGFBP3 genes in spermatogenic cells, and its trend is the same as that in testicular tissue. The effect of Heshouwuyin is better than the three individual active compounds.

### Effects of Heshouwuyin on the insulin/IGF signalling pathway target gene BCL-2 and the cell cycle of spermatogenic cells

**Figure 9.**
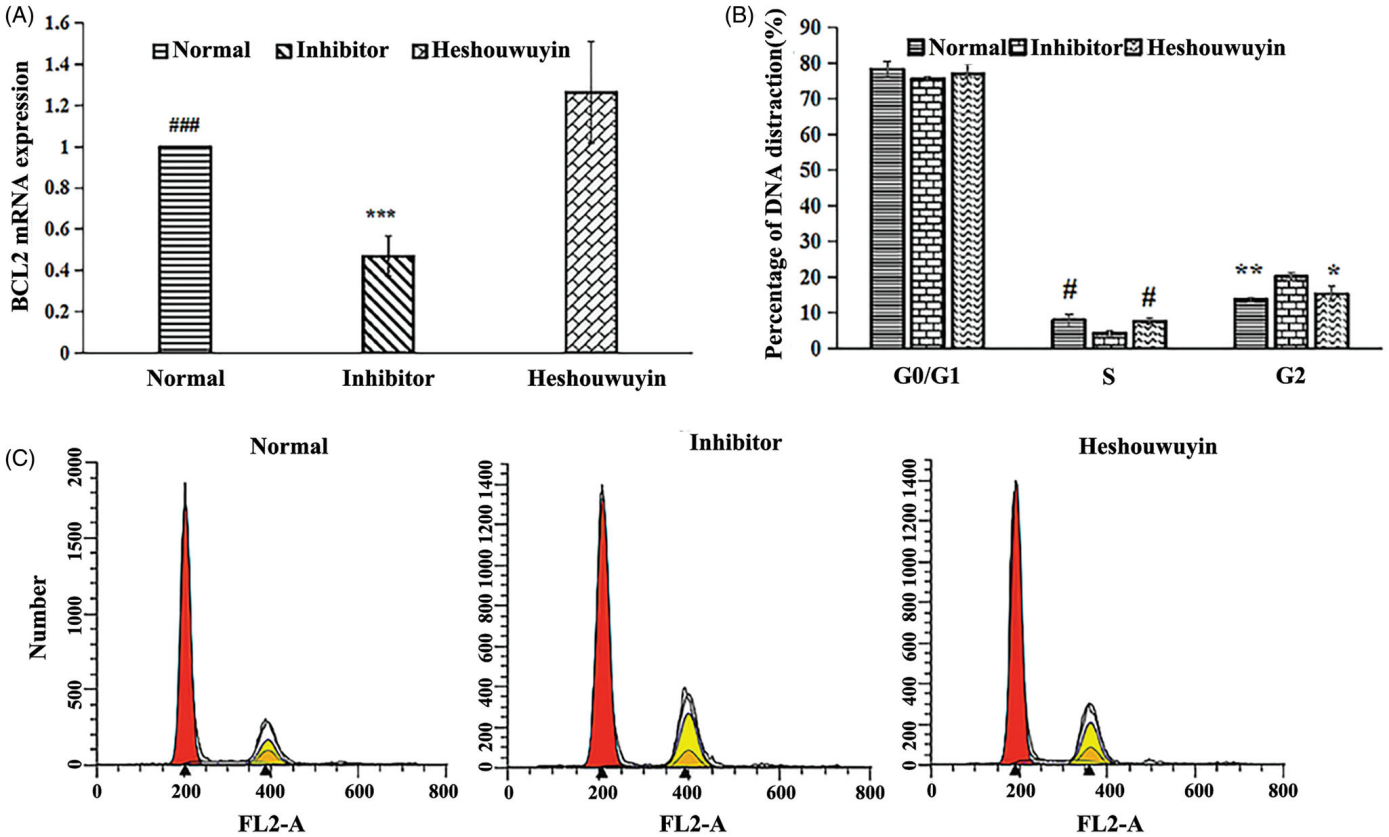
Effects of Heshouwuyin on the insulin/IGF signalling pathway target gene BCL-2 and the cell cycle of spermatogenic cells. (A) Quantitative analysis of the mRNA expression of Bcl-2 in the normal group, inhibitor group and Heshouwuyin group. (B) Flow cytometry analysis of the cell cycle percentage distribution of each group. (C) Quantitative analysis of the percentage of the cell cycle in each group of spermatogenic cells. ###*p* < 0.01: the normal group vs. the inhibitor group; ***p < 0.01: the inhibitor group vs. the Heshouwuyin group; #*p <* 0.05: the normal group and Heshouwuyin group in S phase vs. the inhibitor group; *p < 0.05: the normal group and Heshouwuyin group in G2 phase vs. the inhibitor group; **p < 0.01: the normal group and Heshouwuyin group in G2 phase vs. the inhibitor group (*n* = 3).

BMS-754807, an inhibitor of IGF-1R and INSR, was selected to specifically inhibit IGF-1R and INSR, and the mRNA expression level of BCL-2, a target gene downstream of the insulin/IGF signalling pathway, was detected by qRT-PCR. The results showed that the expression level of BCL-2 mRNA in spermatogenic cells in the inhibitor-treated group was significantly lower than that in the normal group, and the expression level of BCL-2 mRNA in spermatogenic cells in the Heshouwuyin group was significantly higher than that in spermatogenic cells in the inhibitor group (Figure 9(A)). Flow cytometry was used to detect cell cycle changes. The percentage of S-phase spermatogenic cells in the inhibitor group was significantly lower than that in the normal group, and the percentage of G2/M phase cells was significantly higher than that in the normal group. This phenomenon was significantly reversed in the Heshouwuyin group, indicating that Heshouwuyin could reverse the cell cycle arrest induced by BMS-754807 (Figure 9(B,C)).

Our research showed that Heshouwuyin can reverse the inhibitory effect of BMS-754807 on the insulin/IGF signalling pathway.

## Discussion

The Chinese herbal formula Heshouwuyin was optimised from the classical clinical Heshouwu pill used in the *Xuanming prescription* by Hejian Liu, a famous ancient Chinese physician. The Heshouwu pill mainly contains *P. multiflorum*, *C. deserticola*, and *A. bidentata* and has been associated with anti-aging effects and enhancing male reproductive function (Chen et al. 2007). On this basis, *P. cocos, E. brevicornu,* and *S. miltiorrhiza* were added to the ingredients of Heshouwuyin. *Poria cocos, E. brevicornu,* and *S. miltiorrhiza* have the functions of strengthening the spleen and heart (Wang et al. 2013; Zhang et al. 2018), strengthening the kidney and bones (Xu, Xia, et al. 2016; Ma et al. 2018), activating the blood and protecting the liver (Peng et al. 2018; Wang et al. 2019), respectively. Previous studies have shown that the IGF1 content in the serum of aging rats is significantly reduced, and the IGF1 transcription level of interstitial cells and supporting cells in testicular tissues is significantly reduced. Heshouwuyin can reverse the above phenomena (Wang et al. 2011). Moreover, gene chip results showed that the insulin/IGF signalling pathway in testicular tissues of aging rats is regulated by Heshouwuyin.

The IRS protein is a key mediator of insulin and IGF signalling and is involved in the control of metabolic hormones (Copps and White 2012). Phosphorylated INSR and IGF1R activate common substrates for downstream effector networks, including IRS, phosphatidylinositol 3-kinase (PI3K), and mitogen-activated protein kinase pathways (Frezza et al. 2018). Insulin and IGF1 bind and phosphorylate INSR and IGF1R, respectively. Phosphorylated INSR and IGF1R are also involved in the regulation of cell proliferation, differentiation, and survival. The IRS family includes four isoforms, IRS1-4 (Al-Salam and Irwin 2017; Machado-Neto et al. 2018). IRS1 is a mediator of insulin and IGF1, both of which can bind to INSR (Simpson et al. 2017; Frezza et al. 2018). The PI3K/protein kinase B (also known as Akt) signalling pathway is mediated by IRS2 and plays an important role in cell growth, proliferation, and apoptosis (Lei et al. 2018). IGF1 is involved in testicular development during embryogenesis, SC proliferation, and germ cells (GS) proliferation and differentiation (Cannarella et al. 2018).

IGFBP3 is an IGF1-binding protein, binds and inhibits the activity of IGF1, thereby inhibiting the growth and mitosis of peripheral cells and exerting anti-apoptotic effects (Slattery et al. 2004; Cao et al. 2014). The binding capacity of IGFBP3 for IGF1 is significantly higher than that of IGF1R. Once bound, IGFBP3 inhibits the binding of IGF1 to IGF1R and blocks the effects of IGF1 on cells. IGFBP3 also induces the expression of pro-apoptotic proteins Bax and activate the MAPK signalling pathway to promote apoptosis (Cheng et al. 2017). An increased IGF1/IGFBP3 ratio can reduce apoptosis in human testicular tissue and spermatocytes (Lue et al. 2010).

In this study, immunofluorescence, Western blotting, and fluorescence-based quantitative PCR techniques were used and showed that the expression of INSR, IRS1, IRS2, and IGF1 in testicular tissues of aging rats was significantly reduced, the expression of IGFBP3 was significantly increased, the ratio of IGF1/IGFBP3 was significantly decreased, and Heshouwuyin regulated the expression of these genes in the testicular tissues.

To determine whether Heshouwuyin regulates spermatogenesis through the insulin/IGF signalling pathway. We performed experiments with spermatogenic cells. For this purpose, we established a cell senescence model using H_2_O_2_- and FeSO4-generated free radicals to induce cellular damage (Uchida et al. 2017). Using immunofluorescence, Western blotting, and qRT-PCR, we found that Heshouwuyin regulates the expression of INSR, IRS1, IRS2, IGF1, and IGFBP3, which are involved in the insulin/IGF signalling pathway in spermatogenic cells; this regulation likely represents the mechanism involved in the Heshouwuyin-mediated decrease in spermatogenic cell senescence.

We next assessed whether the aging-delaying effect of Heshouwuyin is greater than that of single-compound treatments by treating cells with the individual active herbal compounds contained in Heshouwuyin. Stilbene glycoside, the active compound of *P. multiflorum*, has been shown to improve mitochondrial function, reduce the production of ROS in cells and exert an antiaging effect (Cheng et al. 2018; Ning et al. 2018). Icariin, the active compound of *E. brevicornu*, helps to improve the antioxidant capacity of the sperm and enhance its reproductive function (Sun et al. 2019). Oleanolic acid, the active compound of *C. deserticola*, can alleviate DNA damage and apoptosis of germ cells and restore testicular function in aging rats (Zhao et al. 2017). In our *in vitro* experiments, the effect of each individual active compound on the expression of key genes in spermatogenic and Leydig cells was not as robust as that of Heshouwuyin, although certain effects were similar to those of Heshouwuyin. We conclude that Heshouwuyin exerts a greater effect on the insulin/IGF signalling pathway than the individual active compounds, demonstrating the benefits of the multi-pathway and multi-target approach of traditional Chinese medicine.

When the insulin/IGF signalling pathway is activated, IRS1/2 is activated first, followed by the PI3K/AKT signalling pathway, resulting in the upregulation of Bcl-2 gene expression and regulation of the cell cycle to promote cell proliferation (Uchida et al. 2005; Liu et al. 2010). BMS-754807 is an INSR/IGFR-specific inhibitor. *In vitro*, BMS-754807 (1–10 μM) specifically inhibited INSR/IGFR phosphorylation. Furthermore, it has been shown to inhibit the phosphorylation of downstream substrates of INSR/IGFR and PI3K/AKT and inhibits cell proliferation (Carboni et al. 2009; Kolb et al. 2011). Our results show that the reduction in BCL-2 expression levels and cell cycle arrest in spermatogenic cells induced by INSR/IGFR inhibitors were significantly reversed by Heshouwuyin treatment. This result indicates that in addition to affecting the insulin/IGF signalling pathway, Heshouwuyin may play a role directly by activating the PI3K/AKT signalling pathway. In future studies, we will examine the mechanisms by which Heshouwuyin regulates the insulin-modulated PI3K/AKT signalling pathway.

## Conclusions

The effects of Heshouwuyin on testis tissue and spermatogenic cells show that the formulation regulates the function of testis tissue in aging rats by regulating the insulin/IGF signalling pathway. The results of this study provide an experimental basis for improving the testicular function of aging organisms with traditional Chinese medicine.
